# Supplementary material for: A Model-Based Pharmacokinetic/Pharmacodynamic Analysis of the Combination of Amoxicillin and Monophosphoryl Lipid A Against S. pneumoniae in Mice
Source: Pharmaceutics. 2021 Mar 30;13(4):469. doi: 10.3390/pharmaceutics13040469 (PMC8065677; doi:10.3390/pharmaceutics13040469)
Supplement: Supplementary file 1 [file pharmaceutics-13-00469-s001.pdf]

pharmaceutics

MDPI

Supplementary Materials: A Model-Based Pharmacokinetic/Pharmacodynamic Analysis of the Combination of Amoxicillin and Monophosphoryl Lipid A Against *S. pneumoniae* in Mice

Sebastian Franck, Robin Michelet, Fiordiligie Casilag, Jean-Claude Sirard, Sebastian G. Wicha and Charlotte Kloft

Citation: Franck, S.; Michelet, R.; Casilag, F.; Sirard, J.-C.; Wicha, S.G.; Kloft, C. A Model-Based Pharmacokinetic/Pharmacodynamic Analysis of the Combination of Amoxicillin and Monophosphoryl Lipid A Against *S. Pneumoniae* in Mice. *Pharmaceutics* **2021**, *13*, 469. https://doi.org/10.3390/pharmaceutics13040469

Academic Editor: Victor Mangas Sanjuán & Prof. Dr. Inaki F. Troconiz

Received: 26 February 2021
Accepted: 25 March 2021
Published: 30 March 2021

Publisher's Note: MDPI stays neutral with regard to jurisdictional claims in published maps and institutional affiliations.

Copyright: © 2021 by the authors. Submitted for possible open access publication under the terms and conditions of the Creative Commons Attribution (CC BY) license (http://creativecommons.org/licenses/by/4.0/).

Supplementary Material S1. Preclinical PK and PD Data

A detailed overview of the performed preclinical PK and PD study is reported elsewhere [1]. Briefly, female outbred RjOrl:Swiss (CD-1) and inbred Balb/cJRj mice (6–8 weeks old; body weight: ~25 g (Janvier Labs, Saint Berthevin, France)) were infected intranasally with 1–4 × 10^6 CFU *S. pneumoniae* serotype 1 (clinical isolate E1586, MIC_{AMX} 0.016 mg/L) to cause pneumonia. The mouse model is a robust model of pneumococcal invasive diseases [2–4] and has been used extensively to investigate the antibiotic treatments and the stimulation of the innate immunity by TLR4 agonists [5]. Using inbred and outbred mice also allowed to differ between cohorts being characterized as genetically identical or heterogenous that can be studied in exploratory studies or as representation of variations within a population like humans. The individually assessed and afterwards pooled studies being in line with European regulations and ethical guidelines (Animal facility agreement C59-350009, Institut Pasteur de Lille, APAFIS#5164, protocol 2015121722429127_v4) comprised of PK (AMX concentrations in serum) and PD (bacterial numbers in lung and spleen, survival) investigations differentiating between various study groups (no treatment, monotherapy of either AMX (4 doses in total) or MPLA, combination therapy of AMX and MPLA): 12 h post infection, mice were either not treated or administered intraperitoneally with a standard dose (2.0 mg/kg) of MPLA from *S. minnesota* R595 (Re) TLRpure™ (Innaxon, Tewkesbury, UK), intragastrically by oral gavage with different doses of AMX (Sigma-Aldrich, St. Louis, MO, USA; PK study: 0.4, 14 mg/kg; PD study: 0.2, 0.4, 1.2 mg/kg) or a combination of both drugs (with AMX: PK study: 0.4, 14 mg/kg; PD study: 0.2, 0.4, 1.2 mg/kg). The respective doses were selected based on pre-investigations by Casilag at el. [1] to enable an appropriate immune system stimulation and adequate antibiotic effect in mice. To reliably quantify AMX with concentrations above the lower limit of quantification (LLOQ) in the PK experiments, a comparably high dose of 14.0 mg/kg AMX was selected.

To determine AMX concentrations, serum was collected by retroorbital bleeding with 1–2 samples per individual mouse from in total 106 RjOrl:Swiss mice up to 12 h after antibiotic administration (0.167, 0.5, 1, 2, 3, 6, 12 h). Per time point and study group, 3–4 serum samples were obtained from 3 to 4 individual mice. In the PD study altogether 634 RjOrl:Swiss and Balb/cJRj mice were investigated: At predefined time points after infection and before and after treatment (–12, –8, –4, 0, 1, 2, 6, 12, 18, 24, 36 h), mice were sacrificed, respective organs were harvested and homogenized in 1 mL phosphate buffered saline and viable CFU per lung and spleen, respectively, were determined using standard droplet plate assays with appropriate dilution steps [1]. Depending on the study group, a varying number of time points were investigated per study group (maximum of 10 time points for untreated mice; minimum of one time point (12 h after treatment) for study groups treated with 0.2 mg/kg AMX) with at least 6 mice per time point and study group. To assess the survival of mice in the respective study groups, an additional study was performed to monitor mortality of 196 mice every 24 h over 14 d after infection. Further details of the established murine model and the preclinical study groups can be found in the report by Casilag et al. [1].

Supplementary Material S2. Methods of the PK/PD Model Development and Evaluation

Pharmacometric Analysis

All modelling and simulation tasks were performed using NONMEM® 7.4.1 (ICON Clinical Research LLC, Gaithersburg, MD, USA) which was executed by PsN (version 4.7.0 [6]). As compiler, GFortran (version 6.3.0, GNU Compiler Collection) for macOS High Sierra (Apple Inc., Cupertino, CA, USA) was used. Additional software tools to record, visualize, and evaluate the modelling results were Pirana (version 2.9.7 [7]) including XQuartz (version 2.7.11), RStudio (version 1.2.1335), and R (version 3.6.0) with packages xpose4 (version 4.6.1), ggplot2 (version 3.2.1), and vpc (version 1.1.0). For parameter estimation, Laplacian estimation with interaction and due to stability reasons first-order estimation were used for the pharmacometric PK/PD and TTE model, respectively. To handle samples with concentrations below the LLOQ, the so-called M3 method, which estimates the likelihood that the concentration is below the LLOQ, was used [8].

For comparison between nested or non-nested models, analysis of the objective function value (OFV) or Akaike Information Criterion (AIC) [9], respectively, was performed. A decrease of more than 3.84 ($p \leq 0.05$ at $\alpha = 0.05$, df = 1) of the OFV was defined as statistically significant. In addition, model evaluation was performed by considering goodness-of-fit (GOF) plots, simulation-based diagnostics (e.g., VPC), log-likelihood profiling (LLP) and bootstrap analysis [10]. For the VPC, 1000 simulations including interindividual variability (IIV (PK data)) or unexplained variability (PK/PD data) based on the original dataset were performed resulting in a 90% prediction interval and a 90% confidence interval (CI) around the predicted percentiles (5tt, 50th, and 95th percentile). For final models, a nonparametric bootstrap analysis with 1000 bootstrap samples with replacement stratified to the respective study groups was performed to determine the precision and accuracy (95% CI) of the bootstrap parameter estimates [11]. These were compared to the final model-predicted parameter estimates of the original dataset.

Pharmacokinetic Submodel

For AMX concentrations in serum, one-, two-, and three-compartment models after oral administration with linear and nonlinear absorption and elimination processes and with or without lag time were investigated. Potential covariates such as mouse type (RjOrl:Swiss, Balb/cJRj), MPLA coadministration (yes/no) and the dose of AMX (0.4 mg/kg, 14 mg/kg) were investigated: Their influence on the model parameters clearance, volume of distribution, intercompartmental clearance or absorption rate constant as categorial (mouse type, MPLA coadministration) or continuous (AMX dose) covariate was evaluated. The influence of MPLA coadministration on certain model parameters was evaluated by allowing MPLA as binary covariate to change respective PK model parameters proportionally with the AMX dose:

$$P = \theta_1 + \theta_2 \cdot DOSE \quad (1)$$

where θ_1 is the typical model predicted value of a PK parameter P in mice that were not treated with MPLA and θ_2 is a value for the influence altering θ_1 in case of MPLA coadministration and also depending on the dose of AMX ("DOSE"). Sampling twice per individual mouse due to the developed highly sensitive and little sample volume requiring bioanalytical method, enabled estimation of IIV for the PK data. This source of variability was included for various parameters in an exponential stochastical submodel, assuming PK parameters to be lognormally distributed. The RUV characterising, e.g., experimental imprecision, e.g., in the bioanalytical method and blood sampling was investigated as additive, proportional and combined RUV model.

Bacterial Disease Submodel

To describe the growth of bacteria in untreated mice, a bacterial disease submodel was developed. Based on the visual time course of the bacterial data, the number of bacteria in the lung ($N_{bacteria, lung}$) was characterized by a time-delayed (k_{lag}) first-order growth rate constant (k_g) describing the natural growth of the bacterial strain [12]. To also capture treatment-unrelated killing and natural death processes of bacteria, an additional first-order rate constant ($k_{kill,lung}$) term was introduced [13]:

$$\frac{dN_{bacteria,lung}}{dt} = k_g \cdot \left(1 - \left(e^{-k_{lag}t}\right)\right) \cdot N_{bacteria,lung} - k_{kill,lung} \cdot N_{bacteria,lung} \quad (2)$$

Additional implementations such as limiting bacterial growth to a maximum value or different models, e.g., bacterial killing as E_{max} model [12,13], introducing prebacterial compartments (i.e., bacteria which do not replicate yet) [13] or a simplification without k_{lag}, were investigated and evaluated for improvement in OFV or AIC.

In the spleen, implementation of an analogous growth model as in lung was investigated in detail by applying growth, kill, and death kinetics. The transit process of bacteria from lung to spleen was investigated by transit models being able to delay the transition from one to another comportment more physiologically than simple lag times. The first-order transit rate constant (k_{tr}) was computed by dividing the actual number of transit compartments (n) by the estimated mean transition time (MTT):

$$k_{tr} = \frac{n+1}{MTT} \quad (3)$$

To more specifically estimate the number of transit compartments, an advanced approach was utilized as proposed by Savic et al. [14] using the Stirling approximation.

Effect Compartment Submodel

To link the PK submodel to the time-delayed PD effect of AMX at target sites in lung and spleen, effect compartments were introduced [13,15,16]. Thus, by allowing the transfer of AMX from serum to a lung or spleen compartment, a concentration-dependent time delay was introduced between the respective compartments and different in- and outflow kinetics (first-order, Michaelis Menten) were investigated. By, e.g., using first-order in- and outflow, the concentration in the effect compartment C_e was determined as follows:

$$\frac{dC_e}{dt} = k_{e0} \cdot C_{serum} - k_{e0} \cdot C_e \quad (4)$$

where k_{e0} is a first-order rate constant for the antibiotic transfer and C_{serum} and C_e the concentrations of AMX in serum and the effect compartment, respectively.

Disease and Treatment Submodel

The effect of AMX on certain bacterial disease parameters (k_g, k_{lag}, $k_{kill,lung}$) or as bacterial killing process was characterized by investigating different effect models in the lung and spleen compartment such as a simple linear, a power, an ordinary E_{max} or a sigmoidal E_{max} model: In comparison, the sigmoidal E_{max} model served as reference and the alternative models were evaluated for worsening in the model performance:

$$E(C_e) = \frac{E_{max} \cdot C_e^H}{EC_{50}^H + C_e^H} \quad (5)$$

where $E(C_e)$ is the drug effect of a defined effect compartment antibiotic concentration, E_{max} is the maximum effect, EC_{50} is the AMX concentration to achieve half-maximum effect, C_e is the apparent AMX concentration in either lung or spleen and H is the Hill factor [13].

Since no PK information for MPLA was collected and only one dose of MPLA was investigated, the influence of MPLA treatment was analyzed as binary covariate (yes/no)

and studied for its ability to increase the killing effect characterized by, e.g., $k_{kill, lung}$ (Equation (2)), mathematically implemented as proportionality factor.

To characterize if MPLA had a significant effect on the efficacy of AMX, the potential of MPLA to modify the effect of AMX was investigated, e.g., by implementing an influence on the maximum effect E_{max} or the concentration of AMX to achieve half of the maximum effect EC_{50} according to the General Pharmacodynamic interaction model proposed by Wicha et al. [17] as fractional change of respective effect parameters. Determined 95% CI of the estimated change parameter were evaluated, where the inclusion of 1 in the 95% CI indicated no significant change.

RUV was estimated and implemented additively on a logarithmic scale. For the pooled studies, implementation of IIV was impossible due to the fact that only one sample was collected for quantification of bacteria per organ from each individual mouse. This setting did not allow to reliably estimate more than one hierarchical level of variability simultaneously.

Time-to-Event Analysis

In order to investigate survival, a TTE analysis was performed based on reported survival of mice. Assuming a specific distribution that is able to describe the risk of observing an event, in this case death, at a specific time point a hazard function can be generated quantifying the risk of a mouse changing from alive to dead [18]. Of several existing parametrisations being used as hazard functions h(t), e.g., constant hazard distribution [19], Gompertz [19,20] and Weibull hazard distribution functions [19,21,22], log-logistic function [23], log-normal function [23,24], or surge function [25], the surge function was chosen as the most suited dynamic risk descriptor for the survival data:

$$h(t) = \frac{SA}{\left[\frac{(t-PT)^2}{SW^2}\right]^{\gamma} + 1} \quad (6)$$

where SA is the surge amplitude, PT is the peak time, SW is the surge width at half-maximum intensity, and γ is a shape parameter of the peak.

Variability in the hazard can be explained by potential covariates allowing differentiation between the study groups. Given the hazard function h(t), the survival function S(t) indicating the probability of being alive until a certain study time t can be derived. The same baseline hazard was assumed for all individuals, as only one observation per individual mouse existed due to the nature of the study and no IIV could thus be estimated. Potential covariates were included exponentially as change in hazard on overall survival to generate a link between the PK/PD model and the TTE model [18,26,27]:

$$h_{cov}(t) = h(t) \cdot e^{\beta_1 x_1 + \beta_2 x_2 + .. + \beta_n x_n} \quad (7)$$

where β_i (i=1, …, n) are coefficients characterising the extent of potential covariate effects of different covariate values x_i (i=1, …, n). As PK predictors, mono- or combination therapy, the dose of AMX, C_{max} of AMX, the area under the concentration-time curve (AUC_{AMX}) of AMX, or the actual AMX concentration at a certain time t (C(t)) and respective combinations of single covariates were investigated. PD predictors included bacterial numbers at 12 h or 36 h in lung or in spleen ($C_{lung, time}$, $C_{spleen, time}$), as well as the area under the bacterial number-time curve in lung or spleen (AUC_{lung}, AUC_{spleen}) or the actual bacterial number at time t (CFU(t)). In addition, PK/PD parameters as $T_{>MIC}$, $T_{>EC50}$, the AUC_{AMX} divided by the MIC (AUC_{AMX}/MIC) or C_{max} divided by the MIC (C_{max}/MIC) were evaluated.

Supplementary Material S3. Results of the PK/PD Model

Pharmacokinetic Submodel

A two-compartment model for serum concentrations of AMX (Figure S1), parameterized in terms of clearance (CL_{AMX} = 124 mL/h), intercompartmental flow (Q = 71.9 mL/h), volume of distribution of the central (V_c = 15.4 mL), and peripheral (V_P = 50.7 mL) compartment with first-order absorption kinetics (k_a = 5.04 h^{-1}) including a lag time (t_{lag} = 0.125 h) as well as first-order elimination kinetics best described the data. Parameter estimates for mice were plausible and are shown in Table S1. Investigation of other compartmental models or nonlinear absorption or elimination kinetics did not improve the model performance. Parameter estimates were estimated with acceptable RSEs (<25.5%) and a proportional RUV model (28.2% coefficient of variation). IIV was included on CL_{AMX} (23.4% CV) and Q (25.7% CV). Prediction-corrected VPCs displayed a good predictive performance of the PK model over the entire investigated time period and also captured fractions of samples that were below the LLOQ (Figure S2) as well as model estimates were within the 95% CI of the bootstrap analysis (Table S1). Still, higher variability was observed at higher AMX concentrations between 0 and 0.5 h after treatment (Figure S2A).

Bacterial Disease Submodel

As described in Figure S1 (right), the final bacterial disease submodel consisted of a time-delay (k_{lag} = 0.0595 h^{-1}), bacterial growth (k_g = 0.477 h^{-1}) and treatment-unrelated killing and natural death effects ($k_{kill,lung}$ = 0.274 h^{-1}) in lung. The initial bacterial number of 6.12 log10 CFU/lung in lung was precisely estimated and in the range with the intranasally administered number of *S. pneumoniae* (≈6 log10 CFU). Here, a simultaneous and at the same time reliable estimation of k_{lag} along with k_g was challenging during the entire development process of the PK/PD model with a higher uncertainty especially for k_{lag} (46.2%). However, given the underlying data of the bacterial growth submodel, simplifications or other implementations were not possible without neglecting important trends of initial killing or delayed growth in the data. Here, all parameters were essential to characterize the bacterial growth kinetics sufficiently, and hence a higher uncertainty was accepted.

The transit of bacteria from lung to spleen was best described by exactly estimating the number of transit compartments leading to a MTT of 42.0 h. Due to the marked delay in transition of bacteria determined in spleen about 3 h after treatment, a high number of compartments (*n* = 23) as well as the concomitant high MTT were plausible, especially considering the different barriers that bacteria had to overcome to transit from lung to spleen. In the spleen, the increase in bacterial numbers was only driven by the inflow from lung leading to accumulation of bacteria. The reliable estimation of bacterial growth as well as natural death and treatment-unrelated killing effects as a first-order killing rate constant $k_{kill,spleen}$ was not supported by the underlying data. Due to the setting of the study (e.g., study period of 48 h), bacteria reducing effects only occurred in presence of at least one drug.

Effect Compartment Submodel

Two separate effect compartments with first-order in- and outflow kinetics allowed a virtual transfer of AMX into lung ($k_{e0,\ lung}$ = 0.125 h^{-1}) and spleen ($k_{e0,\ spleen}$ = 0.0435 h^{-1}), ending up in a delayed increase and slower elimination compared to serum ($t_{1/2,spleen}$ = 15.9 h; $t_{1/2,lung}$ = 5.54 h; $t_{1/2,serum}$ = 0.0861 h) with lower maximum concentrations (Figure S5). Hence, the antibiotic effect lasted over a longer time than AMX was actually above the MIC in serum.

Disease and Treatment Submodel

The effect of AMX on *S. pneumoniae* in the lung was best characterized by a sigmoidal E_{max} model as drug-dependent bacterial killing process. The concentration-effect relationship was steep indicated by the estimated high Hill factor, which was not quantifiable with high precision. Nevertheless, H was significantly higher than 1 as indicated by a LLP (95% confidence interval: 1.96–105) and was fixed to 20 to increase model stability [28]. Contrarily, a power model characterized by a first-order killing rate constant (k_{AMX}) with a separate Hill factor representing the slope of the AMX effect compartment concentration and effect relationship in spleen was most stable and best captured killing effects in spleen. More complex implementations of the AMX effect in spleen, such as a sigmoidal E_{max} model, did not improve the model performance. Comparison of the AMX effect in lung and spleen did not only reveal that different mathematical implementations were better able to capture the effect of AMX in the respective compartments, but also the killing effect was substantially increased in spleen. In addition, no other killing effects were present within the structure of the PK/PD model in case of AMX monotherapy. Whereas in the lung, the effect of MPLA was implemented as its ability to enhance the efficacy of $k_{kill,lung}$, in spleen the MPLA-related killing was estimated as a separate first-order killing rate constant and independent of natural death and treatment-unrelated killing effects. These were not able to be included in the spleen given manifold processes affecting the bacterial numbers in spleen. In case of combination therapy with MPLA neither the implementation of MPLA as influence on the maximum effect of AMX (ΔE_{max}, $p = 0.944$, df = 1, $\alpha = 0.05$) nor on the potency of AMX (ΔEC_{50}, $p = 0.272$, df = 1, $\alpha = 0.05$) revealed a significant change of the OFV in a LRT. In addition, the 95% CI of a LLP included parameter estimates of 1 for ΔE_{max} ($\Delta E_{max} = 1.01$; 95%CI 0.875–1.16) and ΔEC_{50} ($\Delta EC_{50} = 0.772$; 95% CI 0.415–1.23), respectively, and no improvement in GOF plots was observed indicating only additive effects.

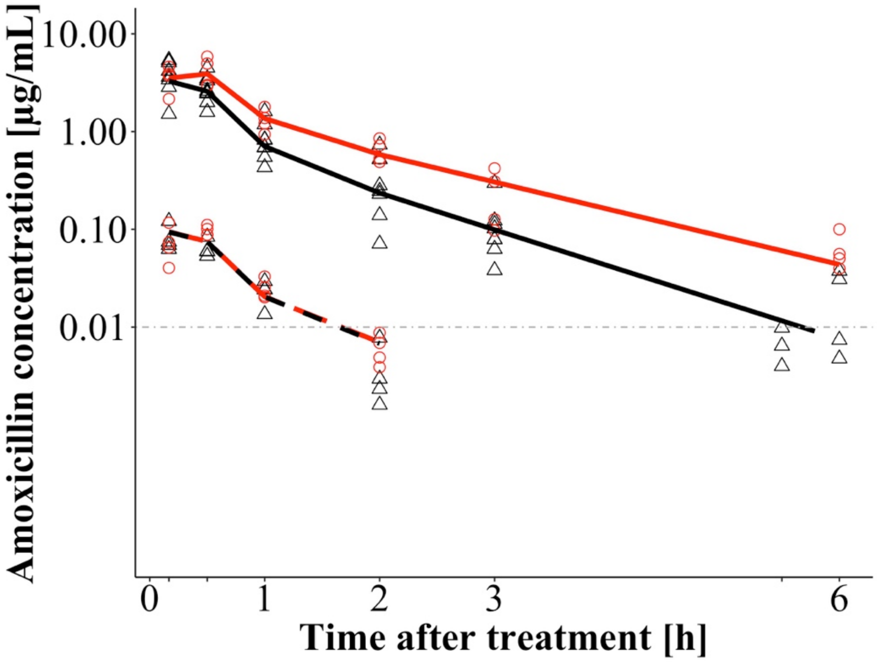

Figure S1. Measured and model-predicted amoxicillin (AMX) concentrations ($n = 106$ with 15.1% below the lower limit of quantification (LLOQ) of 0.01 µg/mL) of the nonlinear mixed-effects submodel for pharmacokinetic data with or without monophosphoryl lipid A (MPLA) coadministration at 0 h. Black triangles: Measurements of AMX monotherapy; Red circles: Measurements of combination therapy of AMX and MPLA; Lines: 50th percentile of model-predicted data after administration of 0.4 mg/kg (dashed) or 14 mg/kg (solid) AMX; LLOQ (horizontal; dot dashed); Colors: AMX monotherapy (black), combination therapy of AMX and MPLA (red).

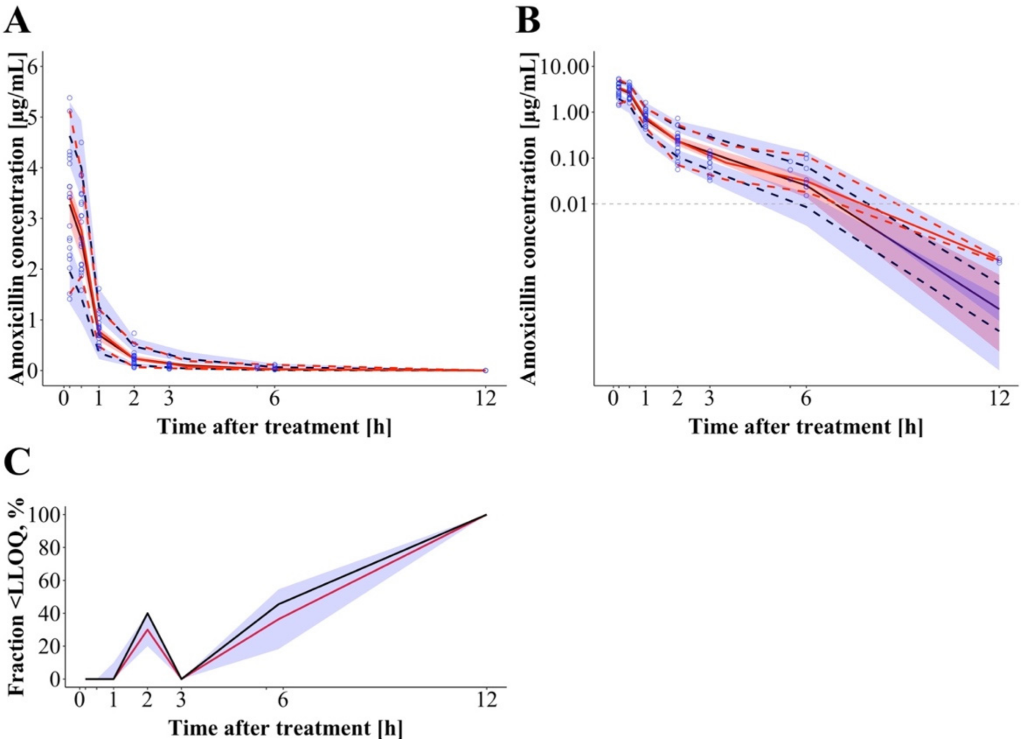

Figure S2. Prediction-corrected visual predictive check (n = 1000 simulations with interindividual variability and unexplained variability) of the developed pharmacokinetic submodel for amoxicillin concentrations (n = 106 with 15.1% below the lower limit of quantification (LLOQ) of 0.01 µg/mL) in mouse serum with or without Monophosphoryl lipid A coadministration in a normal (**A**) and semi-logarithmic (**B**) representation and fraction of samples being below the LLOQ (**C**): Circles: Measurements; Lines: 50th percentile (solid), 5th, and 95th percentile (dashed) of measured (red) and simulated (black) amoxicillin concentrations and LLOQ (horizonal, dashed, grey); Shaded area: 90% confidence interval around simulated percentiles.

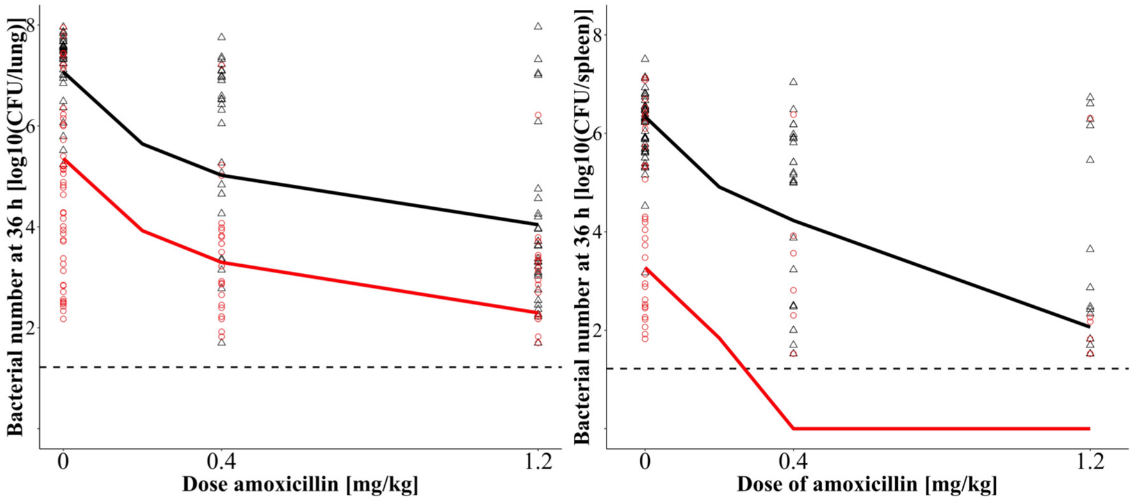

Figure S3. Individual measured (Triangles: Amoxicillin monotherapy, circles: Combination therapy of amoxicillin and monophosphoryl lipid A) and model-predicted (line) bacterial numbers of *Streptococcus pneumoniae* serotype 1 in lung (left) and spleen (right) at 36 h after drug administration of mice treated with different doses of amoxicillin in monotherapy (black) and in a combined treatment with monophosphoryl lipid A (red). The lower limit of quantification is indicated as dashed line.

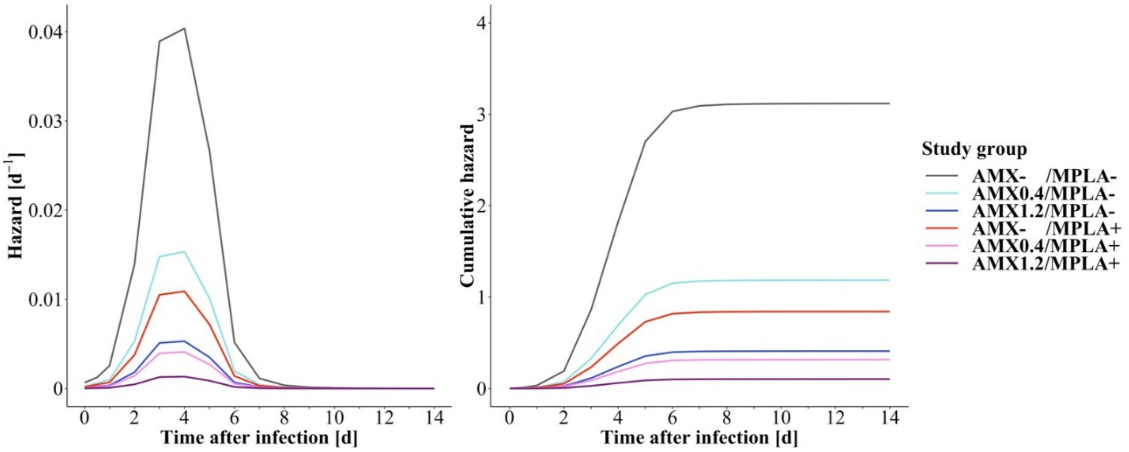

Figure S4. Model-predicted hazard (left) and cumulative hazard (right) in mice being infected with *Streptococcus pneumoniae* serotype 1 and being untreated (AMX-/MPLA-), treated with AMX without (AMX0.4/MPLA-, AMX1.2/MPLA-) or with (AMX0.4/MPLA+, AMX1.2/MPLA+) MPLA coadministration or treated with MPLA (AMX-/MPLA+) administered at 12 h after infection (i.e., t = 0 h in plot) from a time-to-event analysis using a surge function hazard model. Abbreviations: AMX: Amoxicillin (0.40 mg/kg or 1.20 mg/kg); MPLA: Monophosphoryl lipid A (2.00 mg/kg); +: Treatment with respective drug; -: No treatment with respective drug.

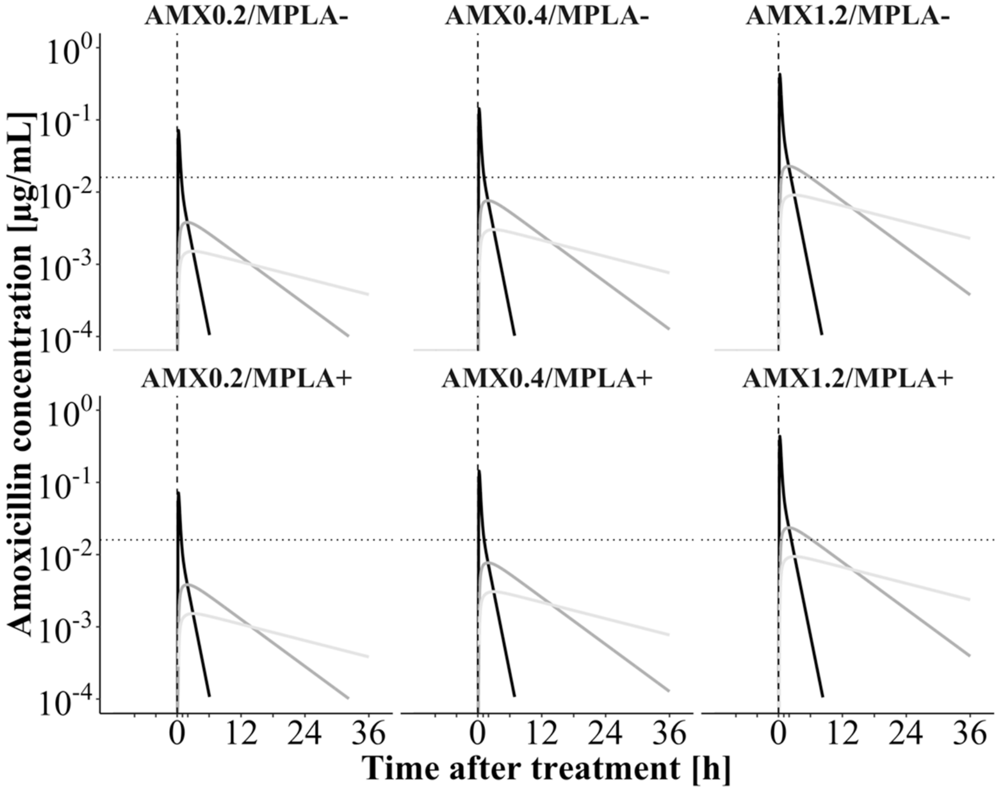

Figure S5. Model-predicted AMX concentrations in the central compartment (black), and respective effect compartments in lung (dark grey) and spleen (light grey) over 36 h after treatment with *Streptococcus pneumoniae* serotype 1 in mice with drug administration at 0 h (dashed vertical line). Different study groups are displayed: Mice treated with AMX (AMX0.2/MPLA-, AMX0.4/MPLA-, AMX1.2/MPLA-) or in a combined treatment (AMX0.2/MPLA+, AMX0.4/MPLA+, AMX1.2/MPLA+) with MIC_{AMX} (dotted horizontal line (0.016µg/mL)). Abbreviations: AMX: Amoxicillin (0.20 mg/kg, 0.40 mg/kg, or 1.20 mg/kg); MIC_{AMX}: Minimal inhibitory concentration of AMX; MPLA: Monophosphoryl lipid A (2.00 mg/kg); +: Treatment with respective drug; -: No treatment with respective drug.

Table S1. Model predicted parameter estimates including bootstrap results (convergence rate of 86.5%) of the pharmacokinetic submodel of amoxicillin and monophosphoryl lipid A.

**Parameter**	**Parameter Estimate**	**Bootstrap**	
**[unit]**	Estimate (%RSE)	Median	95% CI
	**Structural Submodel**		
k_a [h^{-1}]	5.04 *	5.04 *	-
t_{lag} [h]	0.125 (10.0)	0.116	0.0400–0.156
V_c/F [mL]	15.4 (25.5)	17.5	4.41–37.0
V_p/F [mL]	50.7 (10.1)	50.4	38.3–60.6
Q/F [mL/h]	71.9 (17.4)	66.2	33.3–132
CL_{AMX}/F [mL/h]	124 (5.90)	122	108–139
$FC_{AMX+MPLA}$ [mL/h/µg]	–0.145 (18.1)	–0.138	–0.186 to (–0.0958)
	**Interindividual Variability**		
CL, %CV	23.4 (14.3)	21.7	14.9–28.4
Q, %CV	25.7 (43.2)	39.2	8.60–87.1
	**Residual Unexplained Variability**		
Proportional, %CV	28.2	26.5	15.2–33.0

Abbreviations: AMX: Amoxicillin; CI: Confidence interval; CL_{AMX}: Clearance of AMX; CV: Coefficient of variation; F: Bioavailability of AMX fixed to 1; $FC_{AMX+MPLA}$: Fractional change of CL_{AMX} in presence of MPLA depending on the AMX dose implemented as covariate; k_a: First-order absorption rate constant; MPLA: Monophosphoryl lipid A; Q: Intercompartmental clearance; RSE: Relative standard error; t_{lag}: Lag time; V_c: Central volume of distribution; V_p: Peripheral volume of distribution; *Fixed parameter estimate based on model development process (sensitivity analysis, log-likelihood profiling and bootstrap results) for stability reasons.

References

1. Casilag, F.; Franck, S.; Matarazzo, L.; Figeac, M.; Michelet, R.; Kloft, C.; Carnoy, C.; Sirard, J.-C. Boosting Toll-like receptor 4 signaling enhances the therapeutic outcome of antibiotic therapy in pneumococcal pneumonia. *bioRxiv* **2020**, 2020.02.18.955500, doi:10.1101/2020.02.18.955500.
2. Muñoz, N.; Van Maele, L.; Marqués, J.M.; Rial, A.; Sirard, J.-C.; Chabalgoity, J.A. Mucosal administration of flagellin protects mice from Streptococcus pneumoniae lung infection. *Infect. Immun.* **2010**, *78*, 4226–4233, doi:10.1128/IAI.00224-10.
3. Porte, R.; Fougeron, D.; Muñoz-Wolf, N.; Tabareau, J.; Georgel, A.F.; Wallet, F.; Paget, C.; Trottein, F.; Chabalgoity, J.A.; Carnoy, C.; et al. A toll-like receptor 5 agonist improves the efficacy of antibiotics in treatment of primary and influenza virus-associated pneumococcal mouse infections. *Antimicrob. Agents Chemother.* **2015**, *59*, 6064–6072, doi:10.1128/AAC.01210-15.
4. Van Maele, L.; Fougeron, D.; Janot, L.; Didierlaurent, A.; Cayet, D.; Tabareau, J.; Rumbo, M.; Corvo-Chamaillard, S.; Boulenouar, S.; Jeffs, S.; et al. Airway structural cells regulate TLR5-mediated mucosal adjuvant activity. *Mucosal Immunol.* **2014**, *7*, 489–500, doi:10.1038/mi.2013.66.
5. Van Maele, L.; Fougeron, D.; Cayet, D.; Chalon, A.; Piccioli, D.; Collignon, C.; Sirard, J.-C.; Didierlaurent, A.M. Toll-like receptor 4 signaling in hematopoietic-lineage cells contributes to the enhanced activity of the human vaccine adjuvant AS01. *Eur. J. Immunol.* **2019**, *49*, 2134–2145, doi:10.1002/eji.201948234.
6. Lindbom, L.; Pihlgren, P.; Jonsson, N. PsN-Toolkit - A collection of computer intensive statistical methods for non-linear mixed effect modeling using NONMEM. *Comput. Methods Programs Biomed.* **2005**, *79*, 241–257, doi:10.1016/j.cmpb.2005.04.005.
7. Keizer, R.J.; van Benten, M.; Beijnen, J.H.; Schellens, J.H.M.; Huitema, A.D.R. Pirana and PCluster: A modeling environment and cluster infrastructure for NONMEM. *Comput. Methods Programs Biomed.* **2011**, *101*, 72–79, doi:10.1016/j.cmpb.2010.04.018.
8. Beal, S.L. Ways to fit a PK model with some data below the quantification limit. *J. Pharmacokinet. Pharmacodyn.* **2001**, *28*, 481–504, doi:10.1023/A:1012299115260.
9. Akaike, H. A new look at the statistical model identification. *IEEE Trans. Automat. Contr.* **1974**, *19*, 716–723, doi:10.1109/TAC.1974.1100705.
10. Nguyen, T.H.T.; Mouksassi, M.S.; Holford, N.; Al-Huniti, N.; Freedman, I.; Hooker, A.C.; John, J.; Karlsson, M.O.; Mould, D.R.; Perez Ruixo, J.J.; et al. Model evaluation of continuous data pharmacometric models: Metrics and graphics. *CPT Pharmacometrics Syst. Pharmacol.* **2017**, *6*, 87–109, doi:10.1002/psp4.12161.
11. Ette, E.I. Stability and performance of a population pharmacokinetic model. *J. Clin. Pharmacol.* **1997**, *37*, 486–495, doi:10.1002/j.1552-4604.1997.tb04326.x.
12. Rathi, C.; Lee, R.E.; Meibohm, B. Translational PK/PD of anti-infective therapeutics. *Drug Discov Today Technol.* **2016**, *21–22*, 41–49, doi:10.1126/science.1249098.Sleep.
13. Nielsen, E.I.; Friberg, L.E. Pharmacokinetic-pharmacodynamic modeling of antibacterial drugs. *Pharmacol. Rev.* **2013**, *65*, 1053–1090, doi:10.1124/pr.111.005769.
14. Savic, R.M.; Jonker, D.M.; Kerbusch, T.; Karlsson, M.O. Implementation of a transit compartment model for describing drug absorption in pharmacokinetic studies. *J. Pharmacokinet. Pharmacodyn.* **2007**, *34*, 711–726, doi:10.1007/s10928-007-9066-0.

15. Sheiner, L.B.; Stanski, D.R.; Vozeh, S.; Miller, R.D.; Ham, J. Simultaneous modeling of pharmacokinetics and pharmacodynamics: Application to D-tubocurarine. *Clin. Pharmacol. Ther.* **1979**, *25*, 358–371, doi:https://doi.org/10.1002/cpt1979253358.
16. Upton, R.N.; Mould, D.R. Basic concepts in population modeling, simulation, and model-based drug development - Part 3: Introduction to pharmacodynamic modeling methods. *CPT Pharmacometrics Syst. Pharmacol.* **2014**, *3*, e88, doi:10.1038/psp.2013.71.
17. Wicha, S.G.; Chen, C.; Clewe, O.; Simonsson, U.S.H. A general pharmacodynamic interaction model identifies perpetrators and victims in drug interactions. *Nat. Commun.* **2017**, *8*, 2129, doi:10.1038/s41467-017-01929-y.
18. Holford, N. A time to event tutorial for pharmacometricians. *CPT Pharmacometrics Syst. Pharmacol.* **2013**, *2*, e43, doi:10.1038/psp.2013.18.
19. Nurzyńska, K.; Austin, R.P.; Fischer, P.M.; Booth, J.; Gommer, F. Survival of the fittest: Time-to-event modeling of crystallization of amorphous poorly soluble drugs. *J. Pharm. Sci.* **2016**, *105*, 1858–1866, doi:10.1016/j.xphs.2016.03.014.
20. Juul, R.V.; Nyberg, J.; Lund, T.M.; Rasmussen, S.; Kreilgaard, M.; Christrup, L.L.; Simonsson, U.S.H. A pharmacokinetic-pharmacodynamic model of morphine exposure and subsequent morphine consumption in postoperative pain. *Pharm. Res.* **2016**, *33*, 1093–1103, doi:10.1007/s11095-015-1853-5.
21. Hansson, E.K.; Amantea, M.A.; Westwood, P.; Milligan, P.A.; Houk, B.E.; French, J.; Karlsson, M.O.; Friberg, L.E. PKPD modeling of VEGF, sVEGFR-2, sVEGFR-3, and sKIT as predictors of tumor dynamics and overall survival following sunitinib treatment in GIST. *CPT Pharmacometrics Syst. Pharmacol.* **2013**, *2*, e84, doi:10.1038/psp.2013.61.
22. *Pharmacokinetic-pharmacodynamic modeling and simulation*; Bonate, P.L., Ed.; 2nd ed.; Springer Science+Business Media, LLC: New York, 2011; ISBN 978-1-4419-9484-4.
23. Collett, D. *Modelling survival data in medical research*; Collett, D., Ed.; 2nd ed.; Chapman & Hall/crc: London, 2003;
24. Wang, Y.; Sung, C.; Dartois, C.; Ramchandani, R.; Booth, B.P.; Rock, E.; Gobburu, J. Elucidation of relationship between tumor size and survival in non-small-cell lung cancer patients can aid early decision making in clinical drug development. *Clin. Pharmacol. Ther.* **2009**, *86*, 167–174, doi:10.1038/clpt.2009.64.
25. Plan, E.L.; Ma, G.; Nagard, M.; Jensen, J.; Karlsson, M.O. Transient lower esophageal sphincter relaxation pharmacokinetic-pharmacodynamic modeling: Count model and repeated time-to-event model. *J. Pharmacol. Exp. Ther.* **2011**, *339*, 878–885, doi:10.1124/jpet.111.181636.
26. Zufferey, P.J.; Ollier, E.; Delavenne, X.; Laporte, S.; Mismetti, P.; Duffull, S.B. Incidence and risk factors of major bleeding following major orthopaedic surgery with fondaparinux thromboprophylaxis. A time-to-event analysis. *Br. J. Clin. Pharmacol.* **2018**, *84*, 2242–2251, doi:10.1111/bcp.13663.
27. Schindler, E.; Amantea, M.A.; Karlsson, M.O.; Friberg, L.E. PK-PD modeling of individual lesion FDG-PET response to predict overall survival in patients with sunitinib-treated gastrointestinal stromal tumor. *CPT Pharmacometrics Syst. Pharmacol.* **2016**, *5*, 173–181, doi:10.1002/psp4.12057.
28. Wicha, S.G.; Huisinga, W.; Kloft, C. Translational pharmacometric evaluation of typical antibiotic broad-spectrum combination therapies against Staphylococcus aureus exploiting in vitro information. *CPT Pharmacometrics Syst. Pharmacol.* **2017**, *6*, 515–522, doi:10.1002/psp4.12197.
